# Effects of acid and phosphate on arsenic solidification in a phosphogypsum-based cement backfill process†

**DOI:** 10.1039/c9ra04624k

**Published:** 2019-09-06

**Authors:** Tubing Yin, Rushi Yang, Jing Du, Ying Shi

**Affiliations:** School of Resources and Safety Engineering, Central South University Changsha Hunan 410083 China shiyingfriend@csu.edu.cn

## Abstract

Phosphogypsum (PG) produced during phosphoric acid production contains significant amounts of arsenic and can potentially cause adverse environmental and health effects. Cement backfill technology is an effective management technique that is used to store PG to prevent such problems. The goal of this paper is to study the influencing factors and mechanism of arsenic stabilization in a PG-based cement backfill process. First, a leaching toxicity test was conducted, which showed that the arsenic concentration in PG batches ranged from 129.1 μg L^−1^ to 407.1 μg L^−1^, which were all far above the standard limit (10 μg L^−1^) set by GB/T 14848-93. In addition, the arsenic content was higher in samples with larger PG particles. Secondly, hydrogen and phosphate ions were added to the backfill to investigate how they influenced arsenic solidification, and the results indicated that phosphate ions, rather than hydrogen ions, delayed the arsenic solidification process. This suggests that controlling the soluble phosphate in PG will help reduce arsenic pollution during backfilling. A toxicity leaching test was carried out after backfill samples were cured for 28 d. All arsenic concentrations were below the standard limit, indicating that the cement backfill technology ensured the long-term solidification and stabilization of arsenic.

## Abstract 2

Phosphogypsum (PG) produced during phosphoric acid production contains significant amounts of arsenic and can potentially cause adverse environmental and health effects.

## Graphical abstract

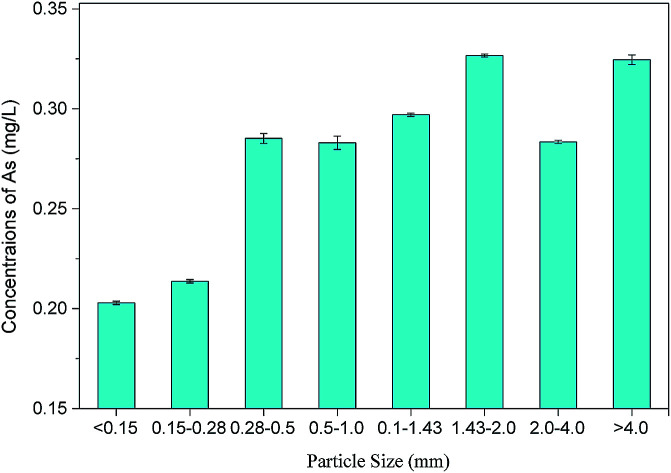

## 1. Introduction

Phosphogypsum (PG) is a solid waste discharged when phosphoric acid is produced *via* a wet process, with 5 tons of PG produced for every ton of phosphoric acid. The annual global production of PG is 100–280 Mt, and China accounts for about 80–85 Mt. PG is primarily composed of CaSO_4_·2H_2_O with some residual phosphoric acid, fluoride, arsenic, and other metals. As a result, PG is usually classified as an acidic by-product with a pH often between 2 and 5.^1^ Water-soluble compounds in PG readily enter water and soil systems after rains, causing serious pollution to surrounding environments.^2^ Arsenic is a highly toxic metalloid in PG that is easily enriched and difficult to remove. Ma *et al.* studied four arsenic-containing gypsum samples using a solid waste-extraction procedure to evaluate its leaching toxicity and showed toxic arsenic leaching concentrations of 8.0–52.5 mg L^−1^.^3^ The lowest concentration was 800 times higher than the limit of national standard of class-II water (arsenic concentration <10 μg L^−1^, GB/T 14848-93), suggesting that untreated gypsum has the potential to release arsenic into the environment. Rafael *et al.* studied how pollutants in PG transformed during water soaking, oxidation, and reduction and found that 2 g of arsenic was released per ton of PG.^4^ This shows that the arsenic in PG, especially the water-soluble part, may pose a serious threat to the environment.

To address PG storage issues, many countries have investigated PG recycling methods, and some recent studies have reported the development of a new method that reuses PG as a filling aggregate during backfilling. When combined with a binder with hydraulic properties, the backfill increased the mechanical performance and durability in underground mines. This created a safe underground working environment and increased the mineral extraction rate.^5–7^ Furthermore, using PG as the backfill aggregate can effectively reduce the surface storing of PG, and it has been estimated that as much as 60% of all produced PG could be consumed using this PG-based backfill technique.^8^ In such a method, however, the backfill is buried underground for many years, which introduces the risk that toxic and harmful substances will secrete and enter groundwater if the PG-based backfill is soaked in it. Therefore, it is important to understand the solidification degree of toxic substances in the backfill. Jiao *et al.* suggested that cemented paste backfill was a safe technology for groundwater, and Uibu *et al.* found that Cd^2+^ and Zn^2+^ could be solidified in a backfilling concrete based on oil shale ash.^9,10^ However, there are few literatures describing the leaching characteristics of arsenic during the backfill process.

Arsenic management has become a major public concern because it can cause acute or chronic toxic reactions in the human body.^11^ Previous studies have proposed several arsenic treatment methods, including extraction, vitrification, and solidification/stabilization (S/S).^12,13^ Among these treatment methods, solidification/stabilization (S/S) has been one of the most effective and broadly-used methods for transforming toxic or potentially hazardous phases into less hazardous ones, especially for waste containing high amounts of arsenic.^14–16^ Liu *et al.* treated arsenic-containing gypsum sludge using S/S, and found that the leached arsenic concentration in the waste decreased from 365.3 mg L^−1^ to 1.36 mg L^−1^.^15^ The S/S treatment also reduced the mobility and bioavailability of arsenic. Shi *et al.* measured arsenic contents in both PG and backfill. Compared with the 50% water-soluble fraction of arsenic in PG, the water-soluble fraction of arsenic in the backfill decreased to 27% after PG inclusion into the backfill matrix.^17^ This was similar to previous study in which the water-soluble arsenic in contaminated sediments decreased by about 20% after S/S treatment.^11^ However, most studies have focused on the leaching amount of arsenic by conducting leaching tests on solidified structures. During the backfill process, there are two possible ways for arsenic to escape the backfilling body. One is through bleeding water due to slurry drainage at the beginning of the backfill hardening process, and the other is through leaching water, which comes from the underground water percolating the hardened backfill body. These two types of water might carry contaminants with them, and may therefore pose an environmental threat.

Arsenic solidification and the factors that influence this process have been the focus of several studies. Coussy *et al.* showed that arsenic dissolution in tailings was affected by temperature, pH, redox conditions, and microorganisms present in mine water.^18^ Hamberg *et al.* showed that arsenic excretion could be reduced by lowering the amount of binder in backfill to reduce water saturation and transform soluble arsenic into stable arsenic.^19–21^ At high proportion of binder in the backfill, reducing the water saturation of the filling body was shown to increase arsenic excretion. Lopes *et al.* added PG to red mud and showed that introducing Ca^2+^ altered the charge balance in the adsorbent, which increased the adsorption of arsenic in red mud.^22^ In PG-based backfill process, Shi *et al.* used BCR sequential extraction to measure the dynamic behaviors of metals in PG-based backfill and found that metals present in backfill were effectively solidified after about 10 years.^17^ However, due to high water-soluble arsenic concentrations, the solidification degree of arsenic should also be considered during backfilling. The arsenic dynamics in both bleeding water and leaching water should also be studied.

Cementitious hydration reactions can change the chemical state of arsenic, while impurities in PG may affect its dissolution behavior. Li *et al.* reported that a decrease in the oxidation state of As (v)–O on the surface of Fe(iii) oxides/hydroxides attributed to the low-alkalinity of the S/S treatment.^23^ Thus the factors affecting arsenic solidification/stabilization (S/S) in backfill should be examined. In this paper, the soluble arsenic content was measured in PG with different particle sizes. Then, considering the residual amounts of phosphoric acid in PG, different amounts of hydrogen ions and phosphate ions were added during the preparation of the backfill slurry, and the arsenic concentrations in slurry bleeding water were measured. The arsenic concentrations as a function of reaction time were also monitored to better understand the S/S process of arsenic in the backfill. Finally, toxicity leaching tests and tank leaching tests were carried out using backfill samples cured for 28 d to study long-term arsenic behavior.

## 2. Materials and experiments

### 2.1 Arsenic content in PG using leaching toxicity test

PG was obtained from Guizhou Kaiyang Group, and nine batches of PG were collected at different storage places to investigate how the arsenic content changed. Leaching toxicity tests of PG were performed according to the method of HJ 557-2010. Deionized water was used as the leaching solution, and 10 g of PG was used for each batch. After mixing deionized water and PG at a liquid–solid ratio of 10 : 1, the mixture was placed on a horizontal vibrator at room temperature and shaken for 8 h at a shaking frequency of 110 times per min. The mixture was then allowed to stand at room temperature for 16 h before being filtered, and the supernatant was collected and stored at 4 °C until further use.

To investigate the influence of particle size on the arsenic content in PG, 600 g of PG was screened with different mesh sizes to obtain eight samples with different particle size ranges (<0.15 mm, 0.15–0.28 mm, 0.28–0.5 mm, 0.5–1.0 mm, 1.0–1.43 mm, 1.43–2.0 mm, 2.0–4.0 mm, and >4.0 mm). The mass fraction of PG in each particle size range was measured by weighing the mass of PG in each group, and then toxicity leaching tests were conducted for each size group.

### 2.2 Preparation of PG-based backfill slurry

The composite binder was obtained by grinding yellow phosphorus slag, cement clinker, fly ash, and quicklime, which has been used in mines for over ten years.^24^ In Batch 1, nine groups of backfill slurries were prepared using PG, binder, and water mixed in a mass ratio of 4 : 1 : 5, which is the same ratio as used in practical applications. The backfill slurry was stirred for 30 min at 200 rpm and then poured into a 40 mm × 40 mm × 40 mm plastic mold. A small hole was drilled in the bottom of each mold to drain excess water in the slurry, which was collected as the bleeding water of the backfill to measure the arsenic concentrations. After draining excess water and the final setting of the backfill, demolding was carried out, and the hardened backfill samples were placed into a chamber at a constant temperature and humidity (20 °C and 95%).

Since phosphoric acid was considered to be the main impurity in PG, hydrogen ions and phosphate ions were added to determine the key factor controlling the S/S process of arsenic. HCl (Sinopharm Chemical, Shanghai, China) was used as the source of hydrogen ions, and a combination of NaH_2_PO_4_ (Sinopharm chemical, Shanghai, China) and Na_2_HPO_4_ (Xilong scientific, Guangdong, China) was used as the phosphate source and also to simultaneously eliminate the impact of pH. The weight of each backfill slurry was 2500 g, and 0.1–1.0 mol hydrogen ions and 0.1–0.5 mol phosphate ions were added to different groups (Table 1).

**Table 1:** Preparation of PG-based cement backfill with addition of hydrogen ions and phosphate ions in Batch 1

Batch 1	*n*(HCl) (mol)	*n*(NaH_2_PO_4_) (mol)	*n*(Na_2_HPO_4_) (mol)
C-1	0	0	0
H-1	0.1	0	0
H-2	0.2	0	0
H-3	0.5	0	0
H-4	1.0	0	0
P-1	0	0.039	0.061
P-2	0	0.078	0.122
P-3	0	0.130	0.203
P-4	0	0.195	0.305

### 2.3 The change in arsenic concentration with time

To explore how the arsenic concentration in backfill slurry changed over time, 9 groups of experiments were performed. As shown in Table 2, the proportions of PG, binder, and additives in Batch 2 were similar to those in Batch 1, but the solid–liquid ratio was 1 : 5 to ensure enough liquid was present in samples. Slurry samples were collected after the slurry was mixed for 5 min, 15 min, 30 min, 1 h, 4 h, 8 h, 24 h, 48 h, 72 h, 96 h, 120 h, and 144 h. Then the slurry was centrifuged at 3000 rpm for 6 min, and the supernatant was filtered and stored at 4 °C to analyze the arsenic concentration.

**Table 2:** Proportion of PG, binder and additives in Batch 2

Batch No.	PG (g)	Binder (g)	*n*(HCl) (mol)	*n*(NaH_2_PO_4_) (mol)	*n*(Na_2_HPO_4_) (mol)
C-2	100	25	0	0	0
H-5	100	25	0.01	0	0
H-6	100	25	0.02	0	0
H-7	100	25	0.05	0	0
H-8	100	25	0.1	0	0
P-5	100	25	0	0.0039	0.0061
P-6	100	25	0	0.0078	0.0122
P-7	100	25	0	0.013	0.0203
P-8	100	25	0	0.0195	0.0305

### 2.4 Toxicity leaching test of backfill

To investigate the leachable arsenic in the backfill, toxicity leaching tests were performed according to the requirements of HJ 557-2010. After curing for 28 days, the backfill samples were grounded into powder, and the toxicity leaching tests were carried out according to the above method.

### 2.5 Tank leaching test

To evaluate the behavior of arsenic in the monolithic backfill, a tank leaching test was performed according to EA NEN 7375:2004. After curing for 28 days, the backfill sample was hung in a tank filled with deionized water. The liquid/solid (L/S) ratio was fixed at 5 cm^3^ water per cm^2^ of exposed solid. After 0.25, 1, 2.25, 4, 9, 16, 36, and 64 days, the leachates were removed, filtered through a 0.45 μm membrane filter, and then stored at 4 °C until arsenic concentration analysis. The tank was recharged with the same volume of fresh deionized water. The mass transfer of arsenic from the backfill was calculated according to the method in Li *et al.*^25^

### 2.6 Measurement methods

#### 2.6.1 Determination of arsenic concentration

To determine the concentration of arsenic, an atomic fluorescence photometer was used according to the following steps.^26^ First, to obtain arsenic standard curves, an arsenic standard solution (0.10 μg mL^−1^) was diluted to create a series of standards with arsenic concentrations of 0.00 μg L^−1^, 1.00 μg L^−1^, 2.00 μg L^−1^, 4.00 μg L^−1^, 8.00 μg L^−1^, and 10.00 μg L^−1^.

A mixed solution of thiourea and ascorbic acid was prepared by dissolving 15 g of thiourea and 15 g of ascorbic acid in 300 mL deionized water, which was used to reduce arsenic to trivalent arsenic in liquid samples, which was further reduced to arsenic hydride using KBH_4_ solution. Using argon gas as the carrier gas, arsenic hydride was decomposed into atoms using a quartz atomizer. The arsenic concentration in liquid samples was determined based on the principle that the atomic fluorescence intensity was proportional to the amounts of elements present in the liquid samples. The detailed arsenic measurement method is shown in the ESI.†

#### 2.6.2 Determination of unconfined compressive strength (UCS) of backfill

Strength is a basic parameter for evaluating a backfill process, and UCS is an inexpensive and easy method to measure the strength development. In this study, after hardened backfill samples were cured for 7, 14, 28, 60, and 90 d, they were removed from the curing chamber for UCS measurements. The UCS tests and calculations were conducted according to the relevant standard JGJ/T 70-2009, using a servo pressure testing machine with 300 kN/10 kN capability (Hualong, China) at a displacement rate of 0.5 mm min^−1^. Each UCS test was conducted in triplicate, and the average values were used for calculations.

#### 2.6.3 Microstructural test

Scanning electron microscopy (SEM) was used to examine the micro morphology of PG and backfill samples (28 d and 90 d).^27^ After the UCS test, the broken backfill was submerged in water-free alcohol to terminate the hydration reaction. Then, the samples were dried and coated with gold to ensure good conductivity. Then microstructures of PG and backfill were obtained using a Helios NanoLab 600i SEM at an accelerating voltage of 10.00 kV and a working distance of 6.0 mm.

## 3. Results and discussion

### 3.1 Particle size distribution of arsenic

Arsenic concentrations in 9 batches of PG leachates ranged from 129.1 μg L^−1^ to 407.1 μg L^−1^, with an average value of 272.1 ± 90.3 μg L^−1^, which was quite similar to arsenic in PG reported in previous studies.^28^ The variation in the arsenic content in PG may be related to the source of phosphate rock and the manufacturing technique. These arsenic leaching concentrations were far lower than the level permitted by GB5085.3-2007 (<5 mg L^−1^), which indicated that the PG used in this study was not a hazardous waste. However, the average leaching concentrations of arsenic were about 27 times higher than the level permitted by GB/T 14848-93 (10 μg L^−1^), indicating that the direct storage of PG might pose serious environmental risks, especially to bodies of water.

**Fig. 1:**
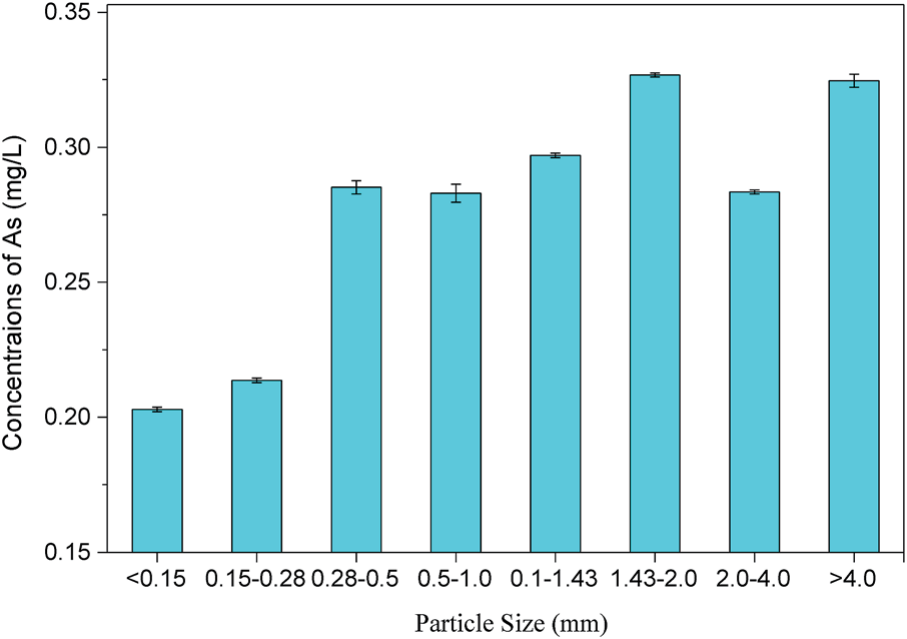
The relationship between particle size and the arsenic concentrations in PG.

The mass fraction of PG in different particle size ranges was determined, and atomic fluorescence spectrophotometry was used to measure the arsenic concentration in toxic leachates of PG within different particle size ranges, as shown in Table 3 and Fig. 1. The results show an uneven distribution of soluble arsenic in PG, with larger particles containing more soluble arsenic. PG particles larger than 4.0 mm accounted for the highest mass fraction (∼35.88%) with an arsenic concentration of 324.6 μg L^−1^ in the leachate, suggesting that PG leaching can be reduced by screening out larger PG particles. This conclusion differs from other studies, such as Al-Hwaiti *et al.* who reported that the arsenic concentration in PG samples showed no significant differences in coarse (>212 μm), medium (53–212 μm), or fine fractions (<53 μm). Thus, it was not necessary for them to distinguish particle sizes when using PG in agriculture.^29^

**Table 3:** PG particle size distribution

Particle size (mm)	<0.15	0.15–0.28	0.28–0.5	0.5–1.0	1.0–1.43	1.43–2.0	2.0–4.0	>4.0
Content (%)	5.52	20.905	5.06	5.995	7.555	4.005	15.08	35.88

### 3.2 Effect of hydrogen ion and phosphate ion on arsenic concentration in backfill slurry bleeding water

**Fig. 2:**
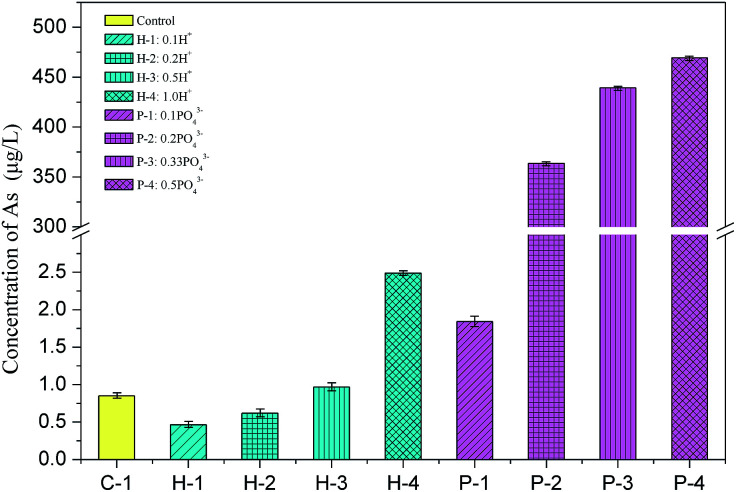
Arsenic concentration in the backfill slurry bleeding water (mixed for 30 min) in Batch 1.

Excess water is usually added to the backfill to maintain the fluidity of the slurry for long-distance transport, which usually drains out in the stopes to form bleeding water. To evaluate whether soluble arsenic escapes with bleeding water, the arsenic concentrations in the bleeding water of Batch 1 was measured and is shown in Fig. 2.

The soluble arsenic concentration in the PG leachate was determined to be 407.1 μg L^−1^. When binder was added, the arsenic concentration significantly decreased to 0.85 μg L^−1^, as shown in sample C-1 in Batch 1. The arsenic solidification efficiency was about 99.8%, indicating that the cementation process effectively solidified arsenic. A similar arsenic S/S efficiency was observed in another study that used cement or fly ash as the binder.^23^ Arsenic excretion in PG may be inhibited by the formation of an insoluble calcium arsenate precipitate, which is then either solidified in the backfill body or encased or adsorbed by hydration products.^30^

The arsenic concentration in the bleeding water was slightly impacted by the amount of added H^+^. Upon addition of 0.1 mol and 0.2 mol of H^+^, the arsenic concentration in the effluent decreased to 0.46 μg L^−1^ and 0.62 μg L^−1^, respectively, indicating a slight inhibition in the arsenic excretion. This slight decrease in the arsenic concentration might be related to the decrease in the hydroxide ion (OH^−^) concentration in the solution since less OH^−^ means it less competition for adsorption locations, increasing the arsenic adsorption efficiency and lowering arsenic secretion.^31^ However, when 1.0 mol H^+^ was added to the backfill slurry, the arsenic concentration in the bleeding water increased to 2.49 μg L^−1^, which was 2.9 times higher than the concentration in the control group. This increase may occur due to an increase in the solution acidity. Fernando *et al.* conducted a BCR sequential extraction test on arsenic-containing tailings and found that the amount of arsenic secreted decreased as the pH decreased when the pH was in the range 3–6.5.^32^ The acidic environment dissolved the calcium arsenate precipitate, which reduced arsenic adsorption and increased arsenic excretion.^32^

Phosphate ions showed a greater influence on arsenic dissolution in backfill slurries by comparing the arsenic concentrations in C-1 group and P-1 to P-4 groups. Fig. 2 shows that the arsenic excretion increased sharply from 1.84 μg L^−1^ to 469.3 μg L^−1^ when the phosphate addition increased from 0.1 to 0.5 mol, indicating that arsenic solidification was strongly inhibited by phosphate. This likely occurred because arsenic is just below phosphorus in the periodic table, and their compounds have similar chemical properties, allowing each of them to combine with calcium ions to form precipitates, but phosphate can effectively compete with arsenate (trivalent) to combine with calcium ions to generate Ca–P compounds which are more insoluble. This interaction between phosphate and arsenic was also studied by Rubinos *et al.* who demonstrated that the arsenic release underwent pronounced kinetic effects, which were strongly influenced by phosphate.^33^ As more phosphate was added, there were fewer adsorption available for arsenate. The arsenic concentration in the bleeding water when 0.5 mol phosphate was added (P-4) was 469.3 μg L^−1^, which was 552 times higher than that of the control group (0.85 μg L^−1^). This concentration greatly exceeded the concentration allowed by the national class-II water standard (<10 μg L^−1^), indicating that this bleeding water cannot be directly discharged into the underground.

The Batch 1 results suggest that the residual phosphoric acid might impact the arsenic S/S process, so it is recommended that its amount be tightly controlled to reduce arsenic discharge in bleeding water. Furthermore, by comparing the results of hydrogen ions and phosphate ions addition, the phosphate ions, rather than hydrogen ions, control the arsenic S/S process. Since phosphates and acids are two common residuals in PG, the arsenic concentrations in the bleeding water should be regularly monitored to avoid contamination.

### 3.3 Relationship between arsenic secretion and time

**Fig. 3:**
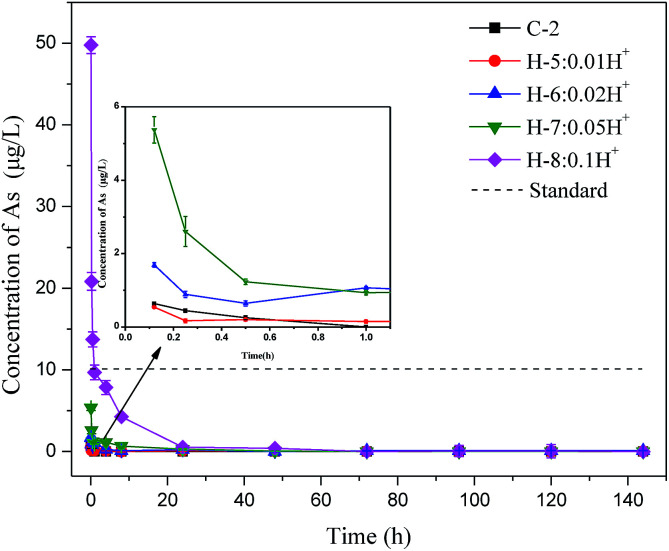
Change in arsenic concentration in control group with time and with H^+^ addition.

To further understand the arsenic S/S process, Batch 2 was used to investigate how the arsenic concentrations varied with mixing time. As shown in Fig. 3, when hydrogen ions were added, the arsenic excretion concentration generally decreased with time, reaching nearly zero after 24 h. The arsenic concentration in the H-8 group with 1.0 mol hydrogen ions exceeded the permitted level within the first 30 min but then decreased rapidly. The arsenic concentrations in all test groups were within the permitted level of the standard after 1 hour.

**Fig. 4:**
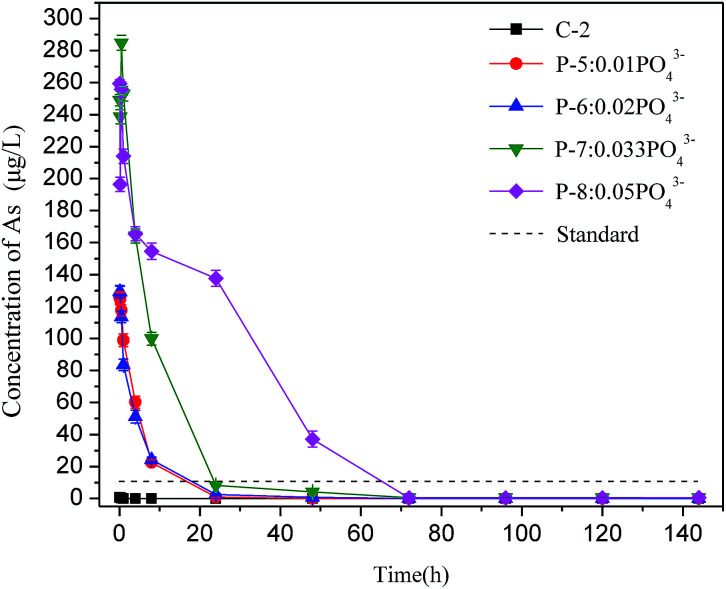
Changes of arsenic concentration in control group and with phosphate addition.

When phosphate ions were added, the soluble arsenic concentration change showed a different trend, as shown in Fig. 4. The arsenic concentrations in the slurry of the four groups (P-5 to P-8) were extremely high in the first 24 hours, with a maximum arsenic concentration of 284.9 μg L^−1^, which was 28 times higher than the level permitted by the national standard. When phosphate was added, the arsenic concentration in the slurry remained nearly constant with no obvious change in the first 30 minutes possibly because the added phosphate preferentially forms sediments. After 30 min, the soluble arsenic concentration started to decrease rapidly, meeting discharge requirement after 72 h. The result of Batch 2 is consistent with Batch 1, which shows again that phosphate is the main factor controlling arsenic concentrations in bleeding water.

In practical application, backfill slurry preparation requires 30 minutes or less before being directly transported to an underground mined-out area through bleeding pipelines. Thus, the arsenic runaway should be controlled when preparing the backfill slurry. The above results show that phosphate impacts the soluble arsenic concentration in bleeding water, especially during the early mixing stage. These results suggest that residual phosphate should be reduced as much as possible during PG production, or that PG be pretreated using modification methods such as water washing to control the phosphate content in the backfill slurry. Such methods would improve the workability of PG and reduce the arsenic concentration in bleeding water so that it meets discharge requirements.

### 3.4 Arsenic leaching of backfill samples after 28 d

**Fig. 5:**
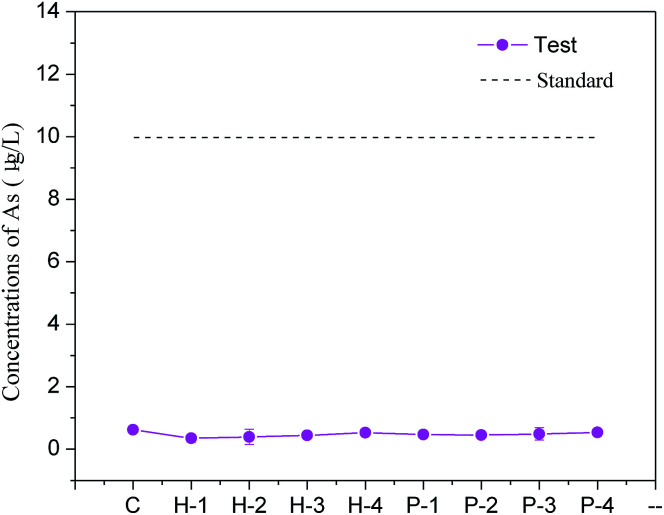
Arsenic concentrations in leachates in backfill samples cured for 28 d.

Backfill samples were cured for 28 d and then subjected to a toxicity leaching test and a tank leaching test to determine the soluble arsenic concentration. The toxicity leaching test in Fig. 5 shows that the arsenic concentration in each test group remained at about 0.50 μg L^−1^, which was far lower than the national class-II water standard. In the tank leaching test, arsenic concentrations in the leaching water were also below the standard (10 μg L^−1^). These results suggest that the use of PG-based cement backfill techniques can successfully solidify arsenic for long-term storage so that it does not seriously pollute groundwater or the environment with arsenic. By analyzing the percolating water in the area using a PG-based cement backfill, Gan *et al.* showed that after years of groundwater leaching, the content of phosphorus, iron, manganese, barium, and other elements could be maintained at relatively low levels.^34^ This indicates that the cement backfill technology can effectively retain most pollutants in PG and reduce environmental pollution compared with PG surface storage.

### 3.5 Strength development of backfill

**Fig. 6:**
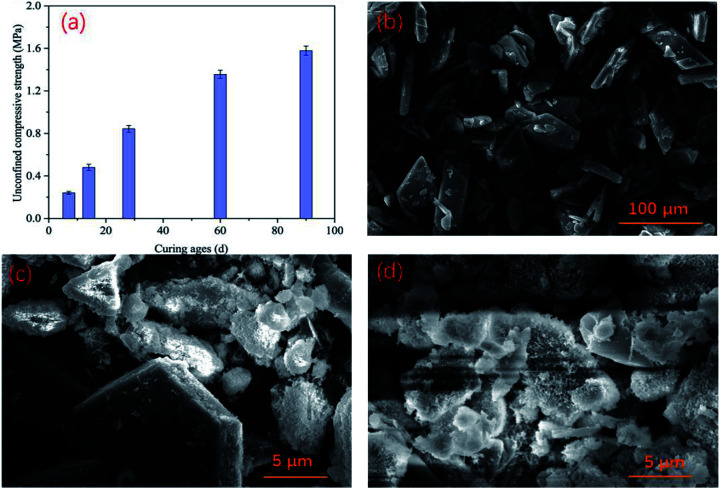
UCS development of PG-based cemented backfill (a), SEM images of PG (b), backfill cured for 28 d (c), and backfill cured for 90 d (d).

After curing backfill in a chamber similar to an underground environment for 7 d, 14 d, 28 d, 60 d, and 90 d, the unconfined compressive strength (UCS) of the backfill in the control group was measured, and the results are shown in Fig. 6(a). The strength of the PG-based cemented backfill increased as the curing time increased, reaching 0.84 MPa after 28 days, which was similar to a previous study.^35^ Furthermore, when the curing age was extended to 90 days, the UCS continued to develop to about 1.58 MPa. The required backfill strength for backfill in mines typically ranged from 0.7–2.0 MPa, so the backfill strength obtained in this study could provide sufficient support in mines. This indicates that the PG-based cemented backfill technology is an applicable mining method.^36^ In this study, even though the PG contained relatively high arsenic contents (407.1 μg L^−1^), the strength development was not obviously affected by such an arsenic level. In addition, the microstructure of backfill was examined *via* SEM in the images shown in Fig. 6. PG showed a plate shape with smooth surfaces, as shown in Fig. 6(b). After being incorporated into the backfill, the binder underwent hydration reactions, and hydration products, such as C–S–H gel and needle-like ettringite, gradually appeared on the PG surface as shown in Fig. 6(c). As the curing time increased, more hydration products were produced (Fig. 6(d)), corresponding to an increase in the UCS. These results could also explain that although PG contained a high arsenic concentration, the hydration reaction could still proceed normally. Furthermore, these hydration products could encapsulate arsenic to a certain extent, thus reducing the mobility of arsenic ions and improving its stability. As a result, the arsenic concentrations in the toxic leachate of the backfill cured for 28 d remained at relatively low levels as discussed above.

## 4. Conclusions

PG contains impurities, so its reuse must consider the dynamics of these impurities to control secondary pollution. To investigate arsenic S/S in the PG-based cement backfill process, the arsenic dynamics in PG, bleeding water, and leaching water, along with the mechanical and microstructural development of the backfill were measured. The results showed that the PG-based cemented backfill technique can better solidify/stabilize arsenic in PG compared with the direct surface storage of PG. To analyze the use of this technique for long-term storage, toxicity leaching tests and tank leaching tests were conducted on hardened backfill cured for 28 d, which showed that arsenic could be stabilized for long periods of time. In addition, the backfill hydration process was not obviously affected by arsenic in PG, and the strength development proceeded well. However, it was found that the arsenic concentration in the backfill bleeding water may exceed the discharge limit. By studying the influence of hydrogen ions and phosphate ions, the phosphate ions, rather than hydrogen ions, were shown to control the arsenic concentration in the bleeding water. In order to utilize more eco-friendly PG in the future, additives are suggested to promote arsenic S/S to ensure the environmental safety of the bleeding water. It is also recommended that the impurities in bleeding water are taken into consideration when recycling the waste as the aggregates during the backfill process of mining.

## Conflicts of interest

There are no conflicts to declare.

## Supplementary Material

RA-009-C9RA04624K-s001
