# Supplementary material for: Effects of acid and phosphate on arsenic solidification in a phosphogypsum-based cement backfill process
Source: RSC Adv. 2019 Sep 6;9(48):28095–101. doi: 10.1039/c9ra04624k (PMC9071007; doi:10.1039/c9ra04624k)
Supplement: RA-009-C9RA04624K-s001 [file RA-009-C9RA04624K-s001.pdf]

Electronic Supplementary Material (ESI) for RSC Advances.
This journal is © The Royal Society of Chemistry 2019

Effects of acid and phosphate on arsenic solidification in phosphogypsum-based cement backfill process

Tubing Yin, [a] Rushi Yang [a], Jing Du [a] and Ying Shi * [a]

[a] School of Resources and Safety Engineering, Central South University, Changsha, Hunan 410083, China.

Supporting Information containing 3 pages, with 2 tables.

1. Determination of arsenic concentration

(1) **Reagents**

- Prepare a series of standard solutions. According to Table S1, a certified arsenic stock solution (1000 μg/mL, National Center of Analysis and Testing for Nonferrous Metals and Electronic Materials GSB04-1714-2004, Beijing, China) was diluted to six working standard solutions with arsenic concentrations of 0.00 μg/L, 1.00 μg/L, 2.00 μg/L, 4.00 μg/L, 8.00 μg/L, and 10.00 μg/L. The mixed solution was prepared by dissolving 15 g of thiourea (Xilong scientific, Guangdong, China) and 15 g of ascorbic acid (Sinopharm Chemical, Shanghai, China) in 300 mL deionized water, which was used to reduce pentavalent arsenic (Ⅴ) to trivalent arsenic (Ⅲ) in liquid samples. As a result, the standard solutions should be standing for about 30 min to reduce all arsenic (Ⅴ) to arsenic (Ⅲ).
- Prepare reductant. The reductant was prepared by dissolving 2 g of NaOH (Guangfu technology, Tianjin, China) and 8 g of KBH_4 (Kermel, Tianjin, China)in 400 mL deionized water. The deionized water was prepared with a laboratory pure water system (Direct-Q3UV, Millipore, USA).
- Prepare carrier fluid. The carrier fluid was 5% of Hydrochloric acid (Sinopharm Chemical, Shanghai, China).

(2) Determinate the arsenic concentration.

A commercial atomic fluorescence spectrophotometer (AFS-2202E, Haiguang, Beijing, China) was used for arsenic quantification, and the instrumental parameters were shown in Table S2. Liquid samples, carrier fluid, and reductant were injected by a peristaltic pump. the volatile arsenic hydride was produced by mixing liquid samples with carrier fluid and reductant. Using argon gas (99.999%, Saizong, Hunan) as the carrier gas, the volatile arsenic hydride was then swept into the flame atomizer. The arsenic concentration in liquid samples was determined based on the principle that the atomic fluorescence intensity was proportional to the amounts of elements present in the liquid samples.

Table S1 Preparation of a series of standards solution

Number	Volume of the 0.10 μg/mL arsenic standard solution (mL)	Volume of 1:1 hydrochloric acid (mL)	Volume of the mixed solution (mL)	Constant volume (mL)	Concentration of standard solution (μg/L)
1	0.00	1	4	10	0.00
2	0.10	1	4	10	1.00

3	0.20	1	4	10	2.00
4	0.40	1	4	10	4.00
5	0.80	1	4	10	8.00
6	1.00	1	4	10	10.00

Table S2 Instrumental parameters of AFS for arsenic measurement

Parameter	Condition
Voltage	-300 V
Lamp current	60 mA
Atomization height	8 mm
Carrier gas flow rate	300 mL/min
Shielding gas flow rate	900 mL/min
